# A novel immersive virtual reality environment for the motor rehabilitation of stroke patients: A feasibility study

**DOI:** 10.3389/frobt.2022.906424

**Published:** 2022-08-29

**Authors:** Giulia Fregna, Nicola Schincaglia, Andrea Baroni, Sofia Straudi, Antonino Casile

**Affiliations:** ^1^ Doctoral Program in Translational Neurosciences and Neurotechnologies, University of Ferrara, Ferrara, Italy; ^2^ Department of Neuroscience and Rehabilitation, Ferrara University Hospital, Ferrara, Italy; ^3^ Department of Neuroscience and Rehabilitation, University of Ferrara, Ferrara, Italy; ^4^ Istituto Italiano di Tecnologia, Center for Translational Neurophysiology of Speech and Communication, Ferrara, Italy

**Keywords:** immersive virtual reality, stroke, motor rehabilitation, head-mount display, fugl-meyer

## Abstract

We designed and implemented an immersive virtual reality (VR) environment for upper limb rehabilitation, which possesses several notable features. First, by exploiting modern computer graphics its can present a variety of scenarios that make the rehabilitation routines challenging yet enjoyable for patients, thus enhancing their adherence to the therapy. Second, immersion in a virtual 3D space allows the patients to execute tasks that are closely related to everyday gestures, thus enhancing the transfer of the acquired motor skills to real-life routines. Third, in addition to the VR environment, we also developed a client app running on a PC that allows to monitor in real-time and remotely the patients’ routines thus paving the way for telerehabilitation scenarios. Here, we report the results of a feasibility study in a cohort of 16 stroke patients. All our patients showed a high degree of comfort in our immersive VR system and they reported very high scores of ownership and agency in embodiment and satisfaction questionnaires. Furthermore, and notably, we found that behavioral performances in our VR tasks correlated with the patients’ clinical scores (Fugl-Meyer scale) and they could thus be used to assess improvements during the rehabilitation program. While further studies are needed, our results clearly support the feasibility and effectiveness of VR-based motor rehabilitation processes.

## 1 Significance statement

Approximately 80% of stroke patients suffer from a hemiparesis of the contralateral upper limb. Motor rehabilitation has been proven to be of key importance to regain, partially or totally, the impaired motor skills. Rehabilitation techniques are based on the repetitive and intense execution of simple motor behaviors. As such they can become taxing and cumbersome for the patients. This often produces non-adherence issues with an obvious negative impact on motor recovery.

Here we describe a novel immersive virtual reality environment for upper limb motor rehabilitation and we report the results that we obtained in a cohort of 16 stroke patients. Our system was designed to turn rehabilitation routines into engaging games and to allow the remote monitoring of the patients’ exercises thus allowing telerehabilitation.

All our patients showed a high degree of comfort in our immersive VR system and they reported very high scores of ownership and agency in embodiment and satisfaction questionnaires. Furthermore, and notably, we found that behavioral performances in our VR tasks correlated with the patients’ clinical scores (Fugl-Meyer scale) and they could thus be used to assess improvements during the rehabilitation program.

## 2 Introduction

Stroke is the second most common cause of death worldwide (Donnan et al., 2008; Feigin et al., 2009) and one of the main causes of acquired adult disability (WHO, 2003; Bonita et al., 2004; Warlow et al., 2008). In most patients, the acute illness produces long-term consequences for them and their families (Langhorne et al., 2011). In particular, brain damage produced by the stroke results in sensory, motor, and cognitive impairments that reduce the patient’s quality of life and social participation (Miller et al., 2010). At the motor level, stroke causes deficits in one of the upper limbs in more than 80% of patients acutely and for more than 40% of them, chronically (Cramer et al., 1997). The sensorimotor recovery of the affected upper limb is a key goal of post-stroke rehabilitation, especially in consideration of its crucial impact on the patient’s independence and quality of life (Pollock et al., 2014). The period immediately following a stroke is critical for regaining, at least partially, motor skills and, if specific rehabilitation programs do not take place there, patients frequently incur in long-term disabilities and reduced quality of life (Patel et al., 2006).

Neurorehabilitation aims at stimulating neuroplasticity after brain injury with the final goal of maximizing motor recovery (Sampaio-Baptista et al., 2018), and it is essential to regain, partially or totally, the impaired motor functions. It has been found that, to achieve best results, motor rehabilitation must be based on repetitive and intensive tasks (Sampaio-Baptista et al., 2018). Specifically, the execution of repetitive task training, executed in sessions repeated several times per week over several weeks, has been proven to be instrumental to increase upper limb functions in stroke patients (Veerbeek et al., 2014). Furthermore, good rehabilitation outcomes seem to be strongly and positively associated with the patient’s motivation and engagement (Langhorne et al., 2011). However, due to its very repetitive nature, neurorehabilitation can quickly become cumbersome for the patients and thus produce severe adherence issues, which negatively affect the rehabilitation outcome (Paolucci et al., 2012). It is thus of outmost importance to develop enjoyable yet clinically effective training procedures.

Gamification procedures have been proposed to make the tasks more entertaining for the patients. However, such “games” are often based on simple tasks executed on a computer screen and thus partially disconnected from everyday gestures and movements. On the contrary, task-specific and context-specific trainings have been proven to be key features for the transferring of the acquired motor skills to real life (Maier et al., 2019a).

All the above issues have been recently further exacerbated by the COVID 19 pandemic that, on the one hand, resulted in a large number of Covid patients needing motor rehabilitation procedures and on the other hand created the need to move out rehabilitation procedures from the hospital to focus the limited clinical resources on the treatment of severe cases.

**FIGURE 1:**
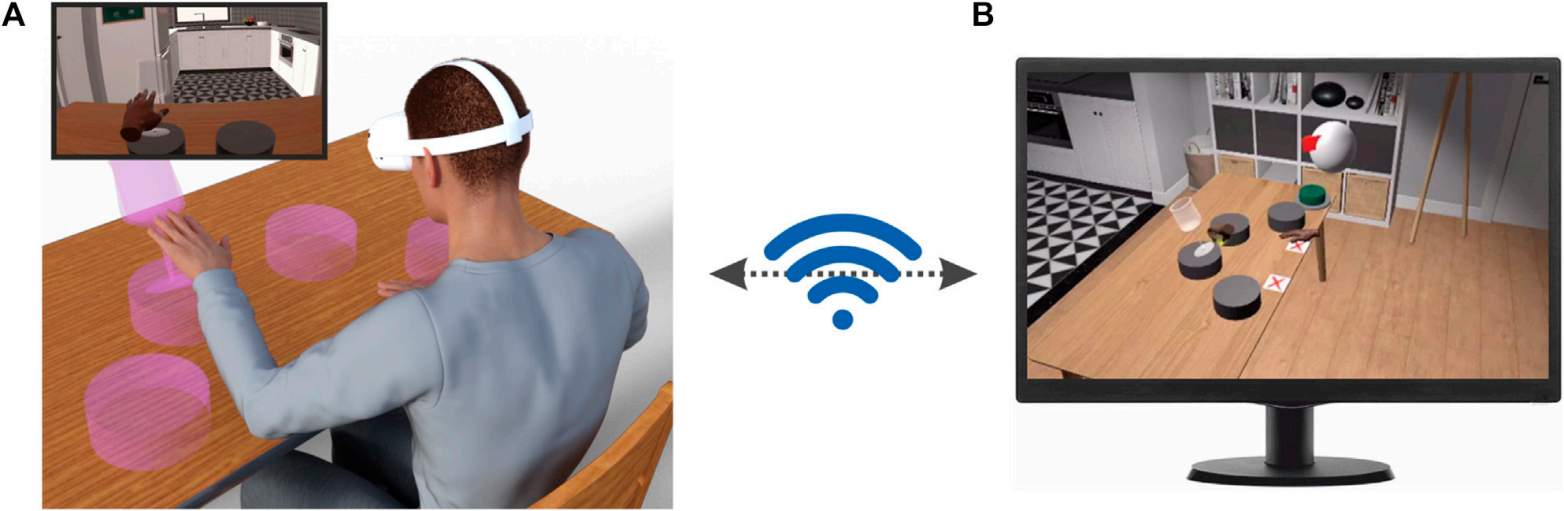
Application scenario of our immersive VR environment. **(A)** The patients are immersed in a VR environment by means of a head-mounted display (HMD, Oculus Quest 2, Facebook Reality Labs). In this environment, they can see different objects with which they can interact. The inset shows the scene as experienced by the patient on the HMD display. **(B)** The program running on the HMD wirelessly communicates with a client app running on a PC that allows to monitor remotely and in real-time the patients’ behavior, set their rehabilitation routines and vocally interact with them.

To address these problems, we leveraged the power of modern computer graphics to design and implement an immersive virtual reality (henceforth VR) environment for upper limb rehabilitation (Figure 1). Immersive virtual reality aims at presenting an artificial environment that replaces the user’s real-world surroundings so as to elicit a convincing perception of “being real”. To this end, the virtual environment has to produce strong illusions of presence (i.e., the feeling of “being there” in the virtual scenario), plausibility (i.e., the feeling that events in the virtual environment are “really happening”), and embodiment (i.e., the feeling that the body the user has in the virtual environment is “really” hers/his) (Slater, 2009, 2018; Slater and Sanchez-Vives, 2016).

Our immersive VR system solves all the major problems related to motor rehabilitation outlined above. Firstly, by leveraging the intrinsic flexibility of VR-generated environments we can present a variety of scenarios and tasks to the patients and keep them interested and focused on their rehabilitation tasks. Secondly, having the patient immersed in a full 3D environment allows us to create tasks that are closely related to everyday activities (e.g. reaching for a glass of water) thus ensuring a transfer of the acquired motor skills to real life. Thirdly, modern VR head-mounted displays are light-weight and compact and they could be easily used at home by patients. Thus, although we are presently testing our system in a clinical setting, it is already fully compatible with potential future telerehabilitation scenarios. Here, we describe the components of our system and report the results of a feasibility study in a cohort of 16 stroke patients. All our patients showed a high degree of comfort in our immersive VR system and they reported very high scores of ownership and agency in standardized embodiment questionnaires (Gonzalez-Franco and Peck, 2018). Furthermore, we found that behavioral performances in our VR tasks correlated with the patients’ clinical scores and they could thus be used to assess improvements during the rehabilitation program. We discuss these findings in the context of present and future clinical scenarios with an emphasis on telerehabilitation and on the potential combination of our VR environment with robotic devices presently used in rehabilitation procedures.

## 3 Materials and methods

### 3.1 Subjects

16 subacute and chronic post-stroke patients (4 female, mean age 62 ± 9) enrolled from the Rehabilitation Units of the Ferrara University Hospital participated in the experiments. They had a wide range of motor impairments and a diagnosis of first, ischemic or hemorrhagic stroke. No age restrictions were applied but patients affected by severe cognitive impairments or other co-existing clinical conditions were excluded. The clinical protocol and all procedures were approved by the local ethical committee (Comitato Etico di Area Vasta Emilia Centro (CE-AVEC) protocol code 897/2020/Oss/AOUFe approved on 17 Marc^h^ 2021).

### 3.2 Experimental procedures

Prior to the experimental procedure, written, informed consent was obtained from all patients. A clinical evaluation of the upper limb impairment and functioning was performed for all the included patients. All the assessments were conducted by the same trained physical therapist. The upper limb motor recovery was assessed by means of the Fugl-Meyer Assessment - Upper Extremity (FMA-UE) (Fugl-Meyer et al., 1975).

We also collected demographic and clinical information to characterize our cohort of patients with respect to age, sex, stroke type, hemiparesis side, days elapsed from the event and hospitalization type (i.e. inpatient or outpatient).

The results of clinical assessments and patients’ demographics are reported in Supplementary Table S1 in the Supplementary Information.

### 3.3 Embodiment questionnaire

To evaluate the degree of embodiment of the virtual hands during the experiment we used a subset of a standardized questionnaire proposed by Gonzalez-Franco and Peck, (2018). The questionnaire was administered in Italian at the end of the session and it consisted of 6 questions (see Sec 1 in the Supplementary Material). The patients could respond to each question by checking one out of 7 possible choices corresponding to a 7 point Likert scale ranging from -3 to 3, with -3 indicating strong disagreement and 3 indicating strong agreement with the statement.

Following Gonzalez-Franco and Peck, (2018), we computed the Ownership and Agency indices by combining the questionnaire’s scores in the following manner:

1. Ownership (Q1—Q2)—Q3.

2. Agency: Q4 + Q5—Q6.

### 3.4 Satisfaction questionnaire

At the end of each experimental session, we also administered a satisfaction questionnaire (see Sec 2 in the Supplementary Material). The questionnaire was administered in Italian and it consisted of 10 items (see Supplementary Material). To six questions the patients had to respond by means of a 5-point Likert scale (1: not at all; 5: very much). Four questions had multiple-choice responses.

### 3.5 Immersive virtual environment and client app

**FIGURE 2:**
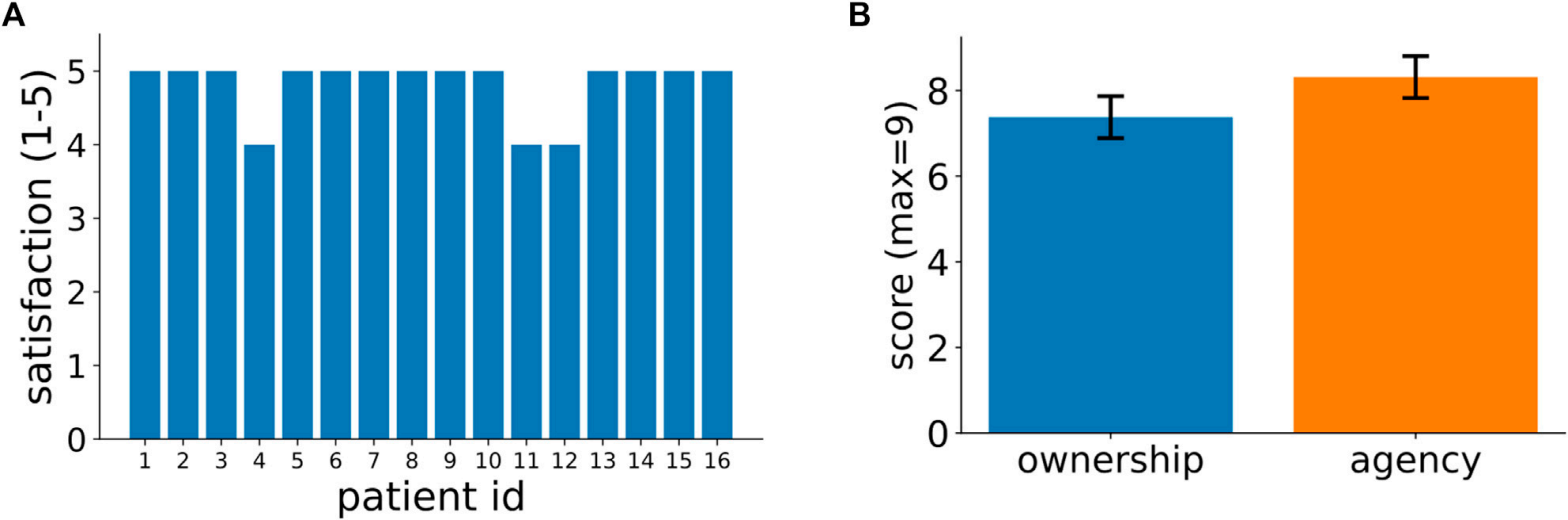
Patients’ feedback on their experience in our VR-based rehabilitation system. **(A)** Patients’ ratings, in a scale from 1 to 5, to the question: “Did you enjoy this type of training?” (In Italian: “Ha gradito la tipologia di allenamento?”). Supplementary Figure S3 in the supplementary information shows the responses for all other questions in the satisfaction questionnaire. **(B)** Patients’ scores for ownership and agency as assessed by a standardized questionnaire (see Methods for further details). The 2 bars represent average across patients and the vertical lines signify variance (mean ownership = 7.4 ± 2.0; mean agency = 8.3 ± 2.0).

Our immersive VR system was developed in C# using the Unity 3D game engine (http://www.unity3d.com). This choice was motivated by Unity’s user-friendliness, easiness of learning, extensive online community and available resources (Kourtesis et al., 2020). Our system consists of two components: (1) A software package installed on the Quest 2 head-mounted display (HMD) that renders the VR environment and manages the execution of the different tasks (Figure 1A) and (2) a client app running under Windows, that wirelessly communicates with the HMD to manage the rehabilitation session (Figure 1B).

As HMD we selected the Oculus Quest 2 for four main reasons. First, it belongs to a new generation of devices that are known to substantially reduce, or even completely eliminate, potential VR induced adverse symptom and effects (VRISE) (Kourtesis et al., 2019a). Second, it has high-end technical specifications (resolution: 1832 × 1920 pixels per eye; refresh rate: 90 Hz; field of view: 90°; head tracking) that support real-time perception and enhanced immersion in virtual scenarios (Slater, 2009). Third, it has on-board capabilities that allow to visually track the patients’ hand movements in real time. Fourth, it is lightweight and price-affordable.

The VR environment consists of a cozy home interior with windows showing a beachside scenarios (Scandinavian Interior Archviz purchased from the Unity Asset Store). This environment was selected based on previous studies suggesting that patients’ motivation during motor rehabilitation is increased by sensory enriched environments containing access to nature and outdoors (Lipson-Smith et al., 2021). Furthermore, it has high graphical quality, a feature that is known to increase the sense of placement in the scene (Slater, 2009). During task execution, the patients sit, both in the real and virtual environments in front of a table (Figure 1A). Notably, the position of the virtual table is registered to that of the real table. During task execution, the patients’ hand and finger movements, visually captured by the Oculus Quest onboard software, are used to animate two virtual hands through which they can interact with virtual objects placed in the scene (e.g. the magenta transparent glass in Figure 1A) to perform different tasks (see below). The virtual hands are displayed from a first-person perspective as it was shown that this point of view is best to elicit a strong sense of embodiment (Slater et al., 2010; Petkova et al., 2011; Maselli and Slater, 2013), potentially due to a stronger activation of the neuronal substrates of action perception (Caggiano et al., 2011; Caggiano et al., 2015; Casile et al., 2011).

During task execution, our system wirelessly communicates with a client app running on a PC that shows a faithful render of the VR environment in which the patients are immersed as well as their virtual hands from a third-person point of view (see Figure 1B for an actual screenshot of the client app). Through this app the rehabilitation therapist can in real-time and remotely monitor the patients’ actions and, in case, vocally interact with them. Furthermore, by means of a pop-up menu (Supplementary Figure S1) the therapist can also manage the rehabilitation session by setting the sequence of tasks and the number of trials per task that the patient has to perform.

Notably, we designed our system such that the HMD and the client app do not need to be on the same local network, thus enabling telerehabilitation scenarios in which the patients can perform most of their routines at home while maintaining strict medical supervision. We performed no detailed technical tests to assess the bandwidth needed for the communication between the HMD and the client app. We tested, however, our VR system in a variety of scenarios ranging from hospital to home networks and even connecting to the internet using a smartphone as hotspot. In all tested conditions, the communication between HMD and the client app ran smoothly. Four tasks are presently implemented in our system, which we called Ball in hole, Cloud, Glasses and Rolling Pin respectively (see Supplementary Figure S2). *Ball in hole*: For this task, a box-like support with a pocket at its center is placed on the virtual table. At the beginning of each trial a tennis ball is placed on this support either to the right or left of the patients and they have to gently push the ball into the hole with their corresponding hand. *Cloud*: At the beginning of trial a cloud of small bubbles appears, which pop upon touching. The cloud is placed either to the right or to the left of the patients and they have to pop all of the bubbles with the corresponding hand. *Glasses*: The task starts with four pedestals presented on the table. The pedestals are distributed along a circle centered on the patient’s body at equal angular displacements (The insets of Supplementary Figure S3,S4 show a simplified view from above of the pedestals). A glass then appears on one randomly selected pedestal and the patients have to push it down (Figure 1A). The patients have to use the hand closer to the pedestal on which the glass appear (two pedestals are closer to the right hand and two are closer to the left hand). *Rolling Pin*: In this task, the patients have to use both hands to move a rolling pin on the table for a pre-defined distance. These four tasks were designed to make the patients execute, in the VR environment, movements that are as close as possible to those usually performed during the rehabilitation sessions.

### 3.6 VR session

Upon coming to the lab, the patient was comfortably seated in a chair in front of a table. The experimenter then helped the patient to wear an HMD and immersed her/him in the VR environment depicting a home interior. In the VR environment, the patient was placed in front of a table as well. The experimenter then used calibration routines programmed in our system to set the height and distance of the table in the VR environment to match those of the real table that the patient was facing. In this manner, when touching the table in the VR environment the patient also experienced a real sensation of touch produced by the real table. This step was implemented, based on previous results showing that the experience of multi-modal (in our case, vision, touch and proprioception) matching cues enhances the feelings of embodiment, presence and immersion of subjects in a VR environment (Gallace et al., 2012; Martin et al., 2022).

The durations of VR sessions for all subjects are reported in Supplementary Table S1 of the Supplementary Materials. The average duration was 50 ± 8.6 min that is within the duration advised in previous work to avoid VRISE (Kourtesis et al., 2019b; Kourtesis et al., 2020), as also confirmed by the complete absence of any reports of adverse effects from our patients.

### 3.7 Correlation analysis

We related behavioral and clinical scores by means of a correlation analysis. To this end, for the subset of 9 patients for which we recorded hand trajectories, we first computed the completion times in the three single-hand tasks (ball in hole, cloud and glasses tasks) as the time difference between when the hand started moving (as obtained from the hand velocity profile) and the trial completion event. For each subject we then computed the difference, between the healthy and the impaired limb, of the median completion times. Finally, for each task, we used a one-tailed Spearman’s rank-order test to correlate these differences with the Fugl-Meyer score across patients. We used a Spearman’s rank-order test as we wanted to investigate the potential presence of correlations with any functional form.

For the glasses task we performed two separate correlation analyses. Indeed, in this task, glasses on pedestals 0 and 3 are closer to the left and right hand respectively compared to the glasses on pedestals 1 and 2. Therefore, completion times were different for glasses on pedestals 0 and 1 (left hand) and 2 and 3 (right hand), as can be appreciated from the distributions shown in SupplementaryFigure S3, S4. To take this into account, we computed two separate distributions: One for the difference in completion times between glasses on pedestals 0 and 3, and one for the different in completion times between glasses on pedestals 1 and 2.

## 4 Results

In the following, we report the results of a feasibility study of our VR system that we performed in a cohort of 16 patients. Each patient was tested once during the performance of multiple consecutive sessions, each consisting of four tasks (see Methods for a complete description of the tasks).

At the end of the experiment, all patients filled in a satisfaction and an embodiment questionnaires (Sec 1 and Sec 2 in the Supplementary Materials). Results in Figure 2A show that almost all patients gave the maximum available score of 5 to the question “Did you enjoy this type of training?”. Similar close-to-maximum ratings were obtained in all other questions of the satisfaction questionnaire (see Supplementary Figure S3). The embodiment questionnaire evaluated the degree of ownership and agency produced by the virtual hands. Both scores range from a theoretical minimum of -9 to a maximum of +9 with positive values indicating increasing levels of embodiments. The average ownership and agency scores across our patients were both very close to their theoretical maximum (mean ownership = 7.4 ± 2.0; mean agency = 8.3 ± 2.0) and significantly different from 0 (ownership: one-sample Wilcoxon test, *p*<<0.001; agency: one-sample Wilcoxon test, *p*<<0.001). In summary, the results of Figure 2 show that our immersive VR system was highly appreciated by the patients and acting by means of virtual hands produced in all of them substantial subjective impressions of ownership of the virtual body and agency.

**FIGURE 3:**
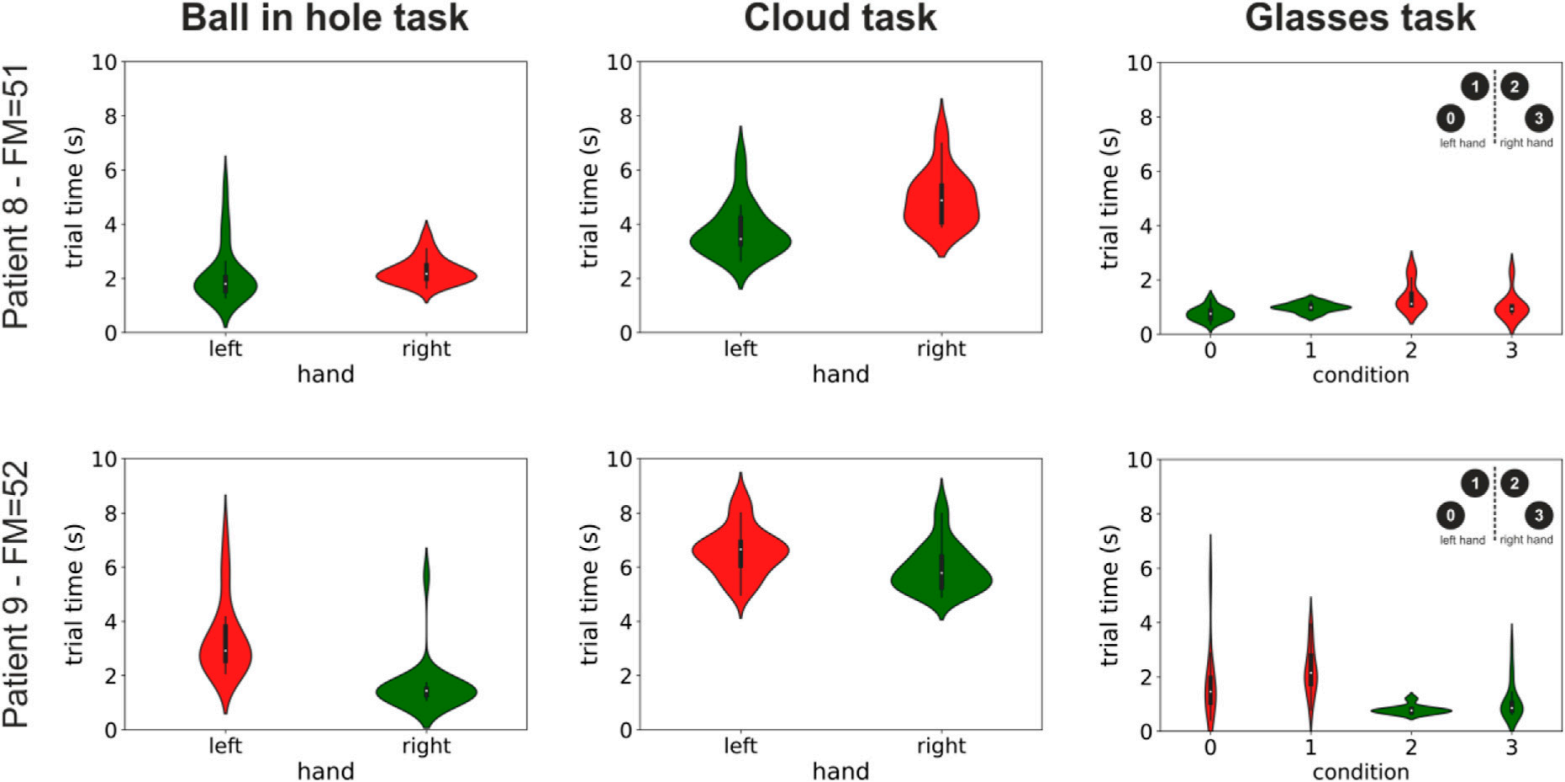
Distribution of completion times for three tasks and two patients. The violin plots show the distributions of the times taken to complete three of the tasks presently implemented in our system (three columns) for two patients. Patient 12 was a 78-year old male with a right-side impairment, and patient 13 was a 63-year old female with a left-side impairment. Distributions are color coded differently for the healthy and impaired limb (green and red respectively). The label on the vertical axis shows the patients’ id and their Fugl-Meyer score. See Supplementary Figure S3 in the supplementary information for similar plots for all other patients.

A very promising use of our environment is that of automatically providing quantitative assessments of motor performance to the therapist to inform the rehabilitation process. This functionality is presently in an initial state and, due to continuous technical development of our system, was available only for a subset of 9 patients. It can nonetheless provide very useful information. To this end, Figure 3 shows the completion times for both the healthy and impaired limb for the three uni-manual tasks presently implemented in our system and for two of our patients: a 78-year old male (patient #12) and a 63-year old female (patient #13). As expected, in almost all conditions, completion times were significantly higher for the impaired compare to the healthy limb (patient 12: Ball in hole task: median left = 1.8s, median right = 2.17s, *p* < 0.01; Cloud task: median left = 3.45s, median right = 4.88s, *p*<<0.01; Glasses task: median condition 0 = 0.76s, median condition 1 = 0.98s, median condition 2 = 1.12s, median condition 3 = 0.93s, p_0,3_ = 0.07, p_1,2_ < 0.01. Patient 13: Ball in hole task: median left = 2.91s, median right = 1.44s, *p*<<0.01; Cloud task: mean left = 6.65 ± 0.88s, median right = 5.79s, *p*<<0.01; Glasses task: median condition 0 = 1.46s, median condition 1 = 2.15s, median condition 2 = 0.77s, median condition 3 = 0.86s, p_0,3_ = 0.023, p_1,2_<<0.001. All *p*-values are from Mann–Whitney U tests). The distributions of task completion times for all other subjects are shown in Supplementary Figure S4.

**FIGURE 4:**
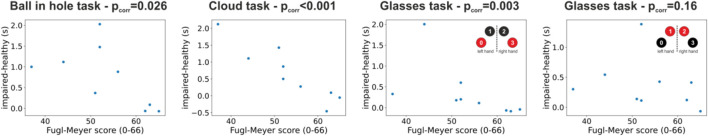
Correlation between behavioral results in our VR tasks and Fugl-Meyer clinical scores. The four scatterplots show the difference in completion times between the impaired and healthy limb for each patient and condition plotted against the Fugl-Meyer clinical assessment. Each panel shows results for one task and each dot represents data for one patients. The *p*-value of the correlation (Spearman’s rank-order correlation) between completion times and Fugl-Meyer scores is shown in the panels’ title.

The results in Figure 3 suggest that completions times could be potentially used to assess the progress during the rehabilitation process. Figure 4 shows the results of a correlation analysis between the differences of the median completion times between the healthy and the impaired limb and the Fugl-Meyer score across our subset of 9 patients The Fugl-Meyer score is one of the most widely used clinical assessment of upper limb motor recovery. It ranges from a minimum of 0 to a maximum of 66, with higher scores indicating less impairment. Very interestingly, we found, even in our necessarily restricted pool of subjects, a significant negative correlation between differences in completion times and clinical scores in almost all conditions (ball in hole task: correlation = -0.66, *p* = 0.026; cloud task = -0.93, *p* < 0.001; glasses task (pedestals 0 3): correlation = -0.82, *p* = 0.003; glasses task (pedestals 1 2): correlation = -0.38, *p* = 0.16; one-tailed Spearman’s rank-order test). The presence of a correlation between behavioral performances in our VR tasks and clinical scores suggests that the former, that are automatically computed by our system, could be conveniently used to measure progress during the rehabilitation process. This result is very promising and it suggests that, in addition to a higher degree of patients’ engagement, our system could also provide, in an automated manner, clinically meaningful indices of motor recovery to the rehabilitation therapists. Further studies in larger cohorts of patients are needed to fully validate this result.

**FIGURE 5:**
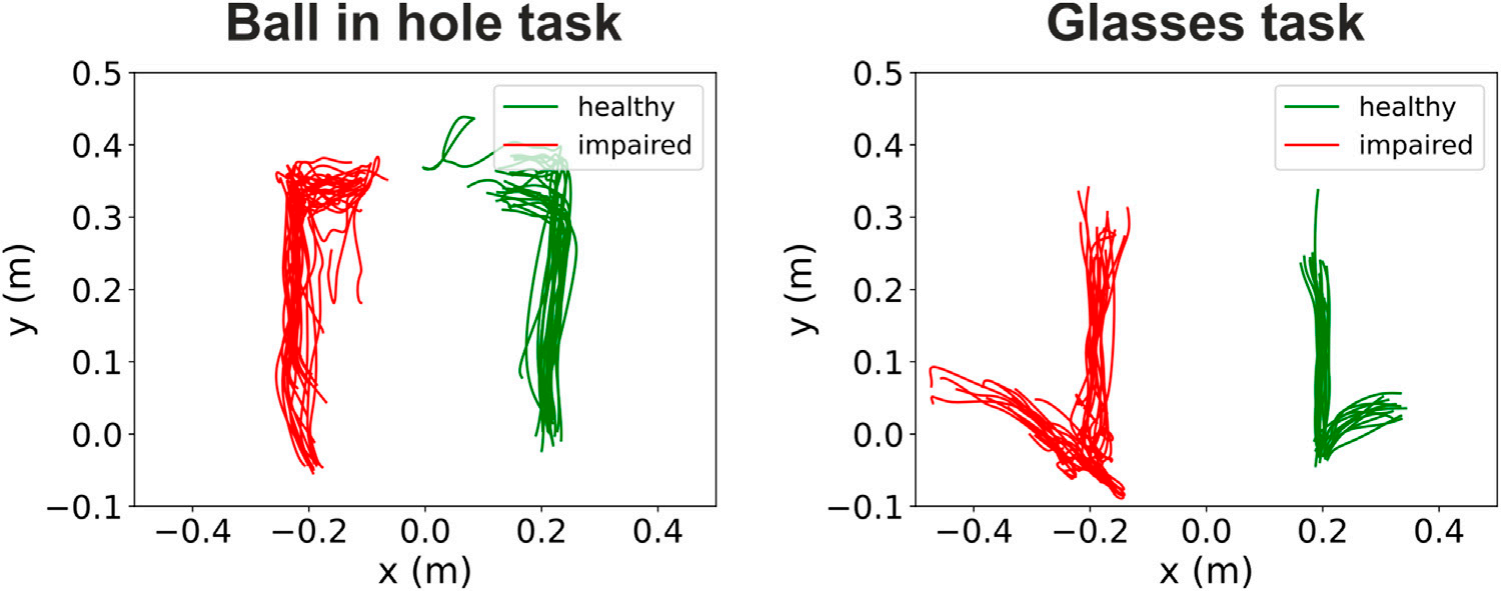
Example of hand trajectories recorded during task execution. The two panels show the hand trajectories of a 77-year old male patient during the execution of the “ball in hole” (left panel) and “glasses” (right panel) tasks. This patient exhibited a left side impairment. The trajectories of the healthy and impaired hands are shown in green and red respectively.

Our system can also automatically store the patients’ hand trajectories during task execution. For example, Figure 5 shows the hand trajectories recorded from a 77-year old male patient during the performance of the Ball in hole (left panel) and Glasses (right panel) tasks. As the figure shows, there are clear differences both in terms of movement span and smoothness between the trajectories of the impaired left arm and the healthy right arm. These trajectories are not presently available to therapists, unless their institution has the availability of an expensive commercial motion capture system. However, they can be easily provided by our VR system. Even their simple visual inspection, presently allowed by our system, can already give therapists relevant information concerning the trajectories of the patients’ arms that can be instrumental to assess the patient’s progress and inform the subsequent steps in the rehabilitation process.

## 5 Discussion

Here, we presented an innovative immersive virtual reality environment for upper limb rehabilitation (Figure 1) and we reported the results of a feasibility study in a group of 16 stroke patients. Almost all subjects gave the maximum rating to their experience (Figure 2A) and, in a standardized questionnaire (Gonzalez-Franco and Peck, 2018), they reported a high degree of ownership of the virtual hands and agency in the VR environment (Figure 2B). Furthermore, we found that behavioral performances in our VR tasks, that can be automatically computed, correlate with the patients’ Fugl-Meyer clinical assessments (Figures 3, 4). This suggests that, in the future, they could be effectively used as an automatically computed proxy of motor recovery. Notably, our system also stores the patients’ hand trajectories. Even a simple visual inspection of these trajectories (see, for example, the plots in Figure 5) can provide valuable clinical information to the expert eye and potentially inform therapeutic decisions. The very positive acceptance of our VR system by patients and the correlation that we found between behavioral performance and clinical scores do suggest that our VR system might represent a very promising direction to expand the toolbox of motor rehabilitation therapists. Furthermore, taken together, our results motivate further studies to explore and validate its clinical efficacy.

A particularly interesting and novel aspect of the present study is that we analyzed the behavioral performances of patients during performance of the VR tasks. This analysis showed that, for almost all of our tasks, the difference in task completion times between the impaired and healthy limb correlated with the Fugl-Meyer score, which is one of the most widely used clinical assessment of upper limb motor functions. This relationship suggests that differences in completion times could be used as a proxy of clinical scores, with two main advantages. First, while the computation of the Fugl-Meyer score requires a non-negligible amount of time and the involvement of specifically trained healthcare professionals, the differences in task completion times can be automatically computed by our system at the end, or even during, each training session. Second, it suggests that task completion times could be potentially used *within* a subject to monitor the efficacy of the rehabilitation process throughout its unfolding in time. We are presently testing this latter point in an ongoing longitudinal study. If these tests will have a positive outcome, then this means that our system could automatically provide ad interim clinically meaningful assessments of the progress of each patient, thus reducing the number of the more time- and resource-consuming clinical assessments. Such a scenario would represent a major step forward, with respect to existing systems, and it would greatly contribute to a more widespread adoption of VR-based motor rehabilitation systems.

It must be emphasized that the goal of our VR system is not to replace current rehabilitation therapies but rather to complement them and strengthen their efficacy (Fang et al., 2022) with a particular focus on two inter-related aspects: enhancing patients’ adherence and provide a viable option for telerehabilitation.

Rehabilitation therapies in post-stroke patients often face adherence issues, in particular due to the need of exercises to be highly intensive and repetitive to effectively induce structural compensatory brain plasticity (Sampaio-Baptista et al., 2018). As such, they often become very tedious for the patients that end up complying only partially, or not at all, with what prescribed by the rehabilitation therapist (K. K. Miller et al., 2017). Gamification procedures have been shown to improve patients’ adherence to the rehabilitation schedules (da Silva Cameirão et al., 2011; Doumas et al., 2021). In this respect, more modern solutions based on immersive VR promise to deliver a more engaging experience to patients producing therefore higher adherence to the prescribed schedules. These solutions are presently gaining increasing traction (Crosbie et al., 2012; Ögün et al., 2019; Mekbib et al., 2021), as recent technical advancements have rendered virtual reality not only extremely realistic but also extremely cost-effective and ready for the consumer market. In addition, clinical studies have proven the effectiveness of these approaches (Laver et al., 2017). Our VR system is based on the Oculus Quest 2 state-of-the-art and off-the-shelf head-mounted display and, as such, it delivers an extremely realistic VR experience at a very accessible cost. In addition, it must be emphasized that, while the Oculus Quest 2 is presently our hardware of choice, the fact that we developed our VR-based rehabilitation system in the Unity development environment using, as much as possible, standard components, ensures that it can be ported to other HMDs with minimal efforts.

Telerehabilitation is a very interesting trend allowed by recent technological advancements. That is, moving part, or even most, of the rehabilitation procedures away from the hospitals, while maintaining medical supervision. Such process has benefits both for the patients and the hospitals. Throughout the rehabilitation period, stroke patients are required to move on a regular basis (i.e. 2-3 times a week) from their houses to a hospital or other healthcare institutions to perform motor rehabilitation sessions under the supervision of trained professionals. That is very taxing for stroke patients, who are motor impaired, and it might produce additional non-adherence issues. Giving stroke patients an effective way to perform certified rehabilitation procedures at home would thus greatly contribute to increase their quality of life. This process would be also beneficial for the hospitals, as it would allow a better management of human and equipment resources, especially in view of handling potential future waves of Covid 19. With this respect, several features of our VR system were specifically implemented to support telerehabilitation scenarios and, as such, they represent a significant advancement with respect to existing immersive VR systems for rehabilitation (Crosbie et al., 2012; Ögün et al., 2019; Mekbib et al., 2021). First, the control app (Figure 1B) communicates with the HMD via the internet. Thus, the computer running the app, controlled by the rehabilitation therapist, and the HMD, wore by the patient, can be in any place with the only requirement that they both have access to the internet. Second, the client app shows an exact replica of what is experienced by the patient in the VR environment. This provides the therapist with real-time information about task performance. Third, the therapist can vocally interact with the patients and set their schedule remotely and in real-time. Fourth, our VR system estimates and stores the patients’ hand trajectories during task performance. As shown in Figures 3–5, these data can potentially give relevant information to the therapist and even provide quantitative and automatic useful indications of how the rehabilitation process is proceeding. In summary, our VR system can not only greatly improve patients’ adherence to prescribed therapies but has been also specifically designed to support telerehabilitation scenarios.

Virtual reality, both immersive and non-immersive, is a mature technique and it is presently experiencing an increasing trend in its adoption for clinical research, psychological interventions and cognitive studies (Blascovich et al., 2002; Lange et al., 2012; Pan et al., 2012; Slater and Sanchez-Vives, 2016; Howard, 2017; Rizzo and Koenig, 2017; Pan and Hamilton, 2018; Krohn et al., 2020; Kourtesis & MacPherson, 2021). Previous studies highlighted the promising role that it might have in the post-stroke rehabilitation of the upper limbs (for review see, for example, Doumas et al., 2021; Laver et al., 2012; Maier et al., 2019b; Mekbib et al., 2020). For example, Mekbib et al. showed that stroke patients undergoing immersive VR-based upper limb motor rehabilitation exhibited a significant increase in Fugl-Mayer score and neural activity in brain areas, particularly those implicated in mirror neurons (Rizzolatti et al., 2014; Casile, 2022), compared to a control group (Mekbib et al., 2021). In a similar fashion, Ögün et al. found a significantly higher increase in several clinical scores in patients undergoing a 6-week immersive VR-based rehabilitation program, compared to a control group (Ögün et al., 2019). Improvements in clinical scores and daily living activities were reported also in studies using non-immersive VR. In a single-group study, Perez-Marcos et al. found significant improvements in clinical scores in chronic patients (i.e. > 6month from stroke) that used a non-immersive embodied VR rehabilitation system for 10 bi-weekly session (Perez-Marcos et al., 2017) and similar outcomes, were also reported by Cameirão et al. in a randomized controlled study (da Silva Cameirão et al., 2011). Results from the present study further support, in agreement with extant literature, the use of VR as a very promising tool in motor rehabilitation. In addition, they also suggest that VR systems, in addition to a clinical outcome, might also provide automatic proxies of clinical scores that (i.e., our results in Figure 4) that can be used by the therapist to take informed decisions during the rehabilitation process.

One potential issue of our VR-based telerehabilitation system is that of privacy. That is, how can one enforce the privacy of the patients’ data when they need to be necessarily transmitted over the internet? This is presently not a real issue for our VR system as it is primarily used for research purposes and information are therefore transmitted only over highly secure clinical networks. In future releases, however, when our system will be deployed in home or non-clinical settings, we plan to enforce privacy in three main ways. First, no personal information will be stored on the HMD and all patients will be identified only by a code. In this manner, the information sent over the internet from the HMD to the client app will be anonymized by design. Second, the match between codes and personal information will be stored on the therapist’s PC and patients’ information on such PCs are already protected by several privacy mechanisms (password protection, encrypted hard disks, firewalls, etc.). Third, a client app can connect to an HMD only if they share a key that is set at compilation time. In this manner, we can off-line and securely set the correspondence between a client app assigned to a given therapist and all the HMDs to which she/he has access.

An additional potential issue that must be addressed by any VR system is that of cybersickness that consists in adverse effects such as nausea and vomiting, postural instability, visual disturbances, or drowsiness caused by the immersion in a virtual space (McCauley & Sharkey, 1992). These adverse effects can have diverse etiologies (Keshavarz et al., 2014; Lawson, 2014; Stanney et al., 2020a) and can strongly limit the adoption of VR-based systems. While earlier HMD elicited cybersickness in a non-negligible percentage of users (Lawson, 2014), reports of cybersickness are not common in modern HMDs and they can be strongly further reduced, and potentially eliminated, by appropriate design choices (Stanney et al., 2020b; Stanney et al., 2021) or subject-specific settings (Stanney et al., 2020a). In our experiments, no patient reported symptoms of cybersickness and the majority of them reported almost no mental or physical fatigue after their VR session (see Supplementary Figure S3). This is likely due to a combination of factors. First, we used the latest generation Oculus Quest 2 HMD that is lightweight, untethered and has a very accommodating design that is known to reduce cybersickness (Stanney et al., 2020b). Second, our patients underwent interactions with the virtual environment of high ecological validity (Parsons, 2015). That is, the virtual hands were controlled in real-time by their own hands; we registered the position of the real table in front of the patient and the table in the virtual space such that the patients experienced a consistent tactile feedback when touching the table in the virtual environment with their virtual hands; many virtual objects exhibited physically-plausible behaviors (e.g., they could be pushed or moved) and we associated veridical sounds to events, where appropriate (e.g., the sound of broken glass in the Glass task). In this manner, the patients experienced, as much as possible, congruent sensorimotor contingencies that are known to increase the illusion of ownership of the virtual body, immersion, presence and plausibility (Slater, 2009; Maselli and Slater, 2013; Slater and Sanchez-Vives, 2016). Finally, our patients had to remain sit throughout the session, which strongly reduced potential visual-vestibular conflicts. These conflicts are one of the causes of cybersickness and are instead more likely during large passive or active bodily movements in a virtual environment (e.g. walking around). That said, patients in the present study were immersed in our virtual environment for the relatively short time of approximately one hour. Further studies, with longer exposures, are thus needed to conclusively exclude the emergence of potential cybersickness issues during usage of our immersive VR system.

One reason for the very positive responses that we obtained from our patients could be the well-known novelty effect. That is, the fact that perceived novelty plays a significant role in the adoption of information technology devices (Wells et al., 2010). We have no evidence either against or in favor of this interpretation. That said, we also believe that this does not represent a limitation either of our study or in the adoption of VR-based rehabilitation systems more in general. Compliance issues are a well-known problem in motor rehabilitation and consistent findings in the literature indicate that the intensity with which the patients execute their rehabilitation routines positively correlates with clinical and functional outcomes (Kwakkel, 2006; Gunnes et al., 2019). Therefore, a VR based systems, as that presented here, that are enthusiastically adopted by patients and that make them perform their assigned routines, or even extra sessions or trials, is, in our opinion, a welcome addition to the therapists’ toolbox, irrespective of the subjective reasons underlying its adoption.

As concerned about future progress of our VR system, the implementation of a mirror modality (that is, a modality in which a virtual hand is animated by the movements of the contralateral real hand) can extend and increase the therapeutic applications in terms of patients’ subgroups and rehabilitative goals. The use of mirror therapy has shown clinical benefits in post-stroke patients in the improvement of upper limb motor function and impairment (Thieme et al., 2018), particularly for severely impaired ones (Colomer et al., 2016; Madhoun et al., 2020). This therapeutic intervention has proven to be instrumental also for pain reduction in patients affected by Complex Regional Pain Syndrome type 1 (Cacchio et al., 2009; Pervane Vural et al., 2016), a frequent and debilitating post-stroke condition that compromises rehabilitative outcomes. The use of immersive VR-based mirror therapy, which is characterized by a more intensive cognitive stimulation, may promote greater effects in these clinical conditions.

While we see many other potential future developments for our VR-based system, a particularly interesting one is its combination with robotic platforms used in motor rehabilitation. These devices are becoming more widespread in the clinical practice and they provide a range of training conditions ranging from the passive resistance to the active assistance of single and multiple body segments during movements (Hesse et al., 2003; Iosa et al., 2012; Mehrholz et al., 2018). Robotic devices are presently routinely used in the clinical practice mainly for gait rehabilitation as they assist in supporting the patient’s bodily weight during training and help leg mobility (Calabrò et al., 2016). Furthermore, it has been shown that the combination of VR and gait-assisting devices enhances the activity of brain networks specifically involved in motor planning and learning (Calabrò et al., 2017). In the past, attempts have been made to combine arm exoskeletons and immersive virtual reality for the upper limb rehabilitation (Frisoli et al., 2009; Frisoli et al., 2007; Montagner et al., 2007). However, potentially due to the bulkiness and cost of exoskeletons, those attempts never translated to the clinical practice. In the past 10 years, robotic devices for upper limb rehabilitation have made consistent progress and they are presently not only used in the clinical practice, but their clinical efficacy has been suggested by several studies (Mehrholz et al., 2018). There are thus presently exciting opportunities for combining them with immersive virtual reality and study whether this combination enhances, similar to the combination of gait training devices and VR, functional brain networks involved in upper limb motor functions.

## Data Availability

The data supporting the conclusions of this article are available from the corresponding author (AC) upon reasonable request.
