# Supplementary material for: A novel immersive virtual reality environment for the motor rehabilitation of stroke patients: A feasibility study
Source: Front Robot AI. 2022 Aug 29;9:906424. doi: 10.3389/frobt.2022.906424 (PMC9465047; doi:10.3389/frobt.2022.906424)
Supplement: Supplementary file 1 [file DataSheet1.PDF]

Supplementary Material

1 Embodiment Questionnaire

The embodiment questionnaire was administered in Italian. It consisted of 6 questions derived from questions Q1, Q2, Q3, Q6, Q7, Q9 in (Gonzalez-Franco & Peck, 2018).

"Durante l'esperimento ci sono stati momenti in cui ...

Q1. Ho avuto la sensazione che le mani virtuali fossero le mie mani"

Q2. Ho avuto la sensazione che le mani virtuali fossero di qualche altro/a"

Q3. Ho avuto la sensazione di avere più di due mani"

Q4. Ho avuto la sensazione di controllare le mani virtuali come se fossero le mie mani"

Q5. I movimenti delle mani virtuali fossero causati da quelli delle mie mani"

Q6. Ho avuto la sensazione che le mani virtuali si muovessero da sole".

English Version:

"During the experiment there were moments in which…

Q1. I felt as the virtual hands were my hands"

Q2 I felt as the virtual hands were someone else's"

Q3. I felt as I had more than two hands"

Q4. I felt as I could my virtual hands as it were my hands"

Q5. I felt as the movements of my virtual hands were caused by my hands"

Q6. I felt as the virtual hands were moving by themselves"

2 Satisfaction Questionnaire

The satisfaction questionnaire was administered in Italian. It included 14 questions investigating pros and cons of the experience of the patients during the VR session. The numbering of the questions reflects the order in which they appeared in the questionnaire. Responses to question S1 are shown in Figure 1A in the main manuscript. Responses to other questions are shown in Figure S3 in the Supplementary Material.

Likert-scores questions (0-5):

S1. Ha gradito la tipologia di allenamento?

S2. I suggerimenti che compaiono durante i giochi sono utili?

S3. Il tempo di allenamento è stato adeguato?

S4. Gli obiettivi dei giochi sono facili da raggiungere?

S5. Le istruzioni sono facili da capire?

S6. Quante pause ha fatto durante la sessione di gioco?

S11. È stanco fisicamente?

S12. È stanco mentalmente?

S13. Quanto si è divertito?

S14. Quanto è soddisfatto della sua prova?

Multiple-choices questions:

S7. In quale compito ha avuto i risultati migliori?

S8. In quale compito ha avuto i risultati peggiori?

S9. Qual è il compito più divertente?

S10. Qual è il compito più noioso?

English Version:

Likert-scores questions:

S1. Did you enjoy the training session?

S2. Were suggestions during the exercise useful?

S3. Was the training length appropriate?

S4. Were the goals task easy to achieve?

S5. Were the instructions easy to understand?

S6. How many breaks did you take during the game session?

S11. Are you physically tired?

S12. Are you mentally tired?

S13. How much fun did you have?

S14. How satisfied with the training session are you?

Multiple-choices questions:

S7. In which task did you perform best?

S8. In which task did you perform worst?

S9. Which task was most enjoyable?

S10. Which task was the most boring?

Supplementary Material

Table S1 - Demographic and clinical characteristics of all patients in our study *(FMA-UE: Fugl Meyer Assessment – Upper Extremity).*

**ID**	**SEX**	**AGE**	**STROKE TYPE**	**HEMIPARESIS SIDE**	**DAYS FROM STROKE EVENT**	**HOSPITALIZATION TYPE**	**FMA-UE score** (0-66)	**VR SESSION** (min)
1	M	61	Hemorrhagic	Left	382	Day-Hospital	43	45
2	M	58	Ischemic	Right	56	Full	34	35
3	M	57	Ischemic	Right	174	Day-Hospital	30	45
4	M	64	Ischemic	Right	66	Full	65	50
5	F	64	Ischemic	Left	106	Day-Hospital	30	45
8	M	56	Ischemic	Left	383	Day-Hospital	35	45
10	F	55	Hemorrhagic	Right	40	Full	60	45
12	M	78	Ischemic	Right	32	Full	51	40
13	F	63	Ischemic	Right	39	Full	52	60
14	M	77	Ischemic	Left	149	Full	37	60
15	M	61	Ischemic	Right	24	Full	65	60
16	M	67	Ischemic	Left	75	Full	44	45
17	M	65	Hemorrhagic	Left	92	Full	63	60
18	M	76	Hemorrhagic	Left	92	Full	52	45
19	M	44	Hemorrhagic	Left	196	Full	62	60
20	F	52	Ischemic	Right	4216	Day-Hospital	56	60

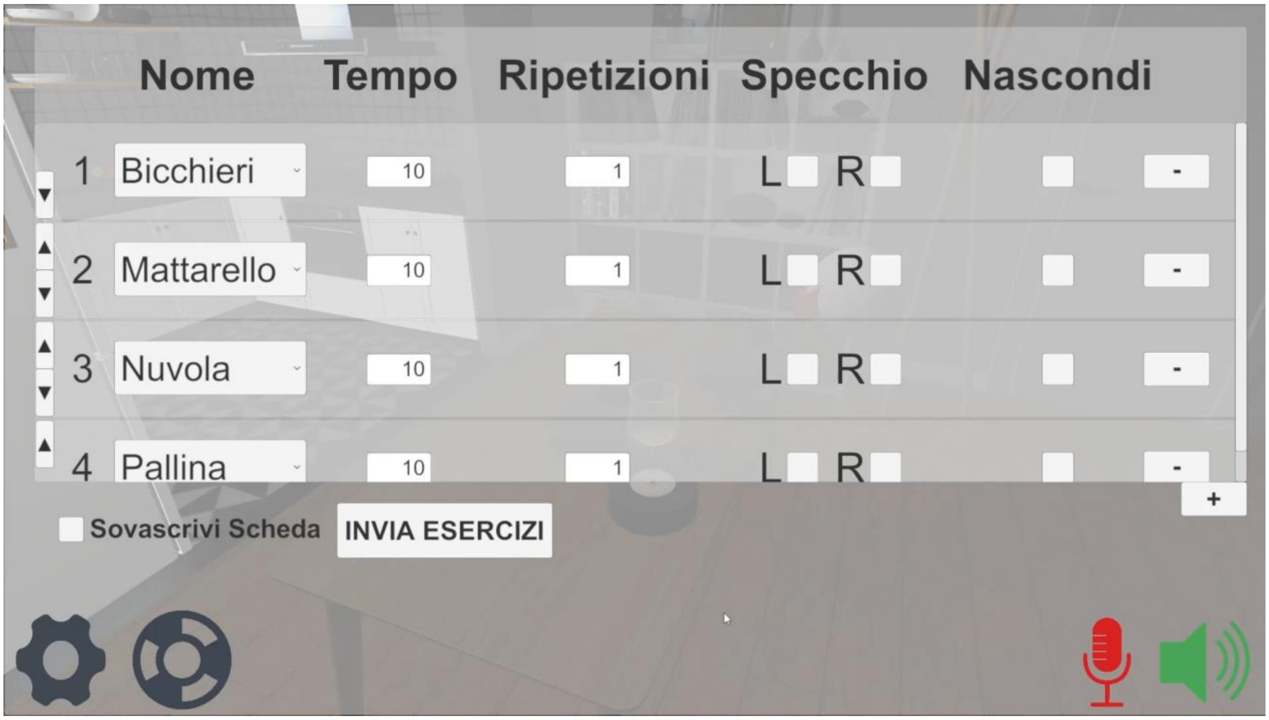

Figure S1 – Menu used to manage the rehabilitation session – Through this menu the rehabilitation therapist can set the sequence of tasks ("Nome" → Name), the timeout for each trial ("Tempo" → Time), the number of repetitions for each task ("Ripetizioni" → Repetitions), whether the movements of one of the two hands are mirrored onto the other ("Specchio" → Mirror) and whether the "mirroring" hand should be displayed or not ("Nascondi" → Hide). A new task is added by clicking on the "+" button in the lower right corner and the task list can be of arbitrary length, which allows multiple repetition of the same task within a session. After building the task list, the therapist can send it to the HMD wore by the patient by clicking on the button "Invia Esercizi" (Send Exercises). The therapist can also in real-time talk and listen to the patient. This functionality is managed by means of the microphone and loudspeaker icons in the lower right corner. Clicking on either icon cycles its color between red and green. When the color is green the corresponding functionality (talking or listening) is enabled.

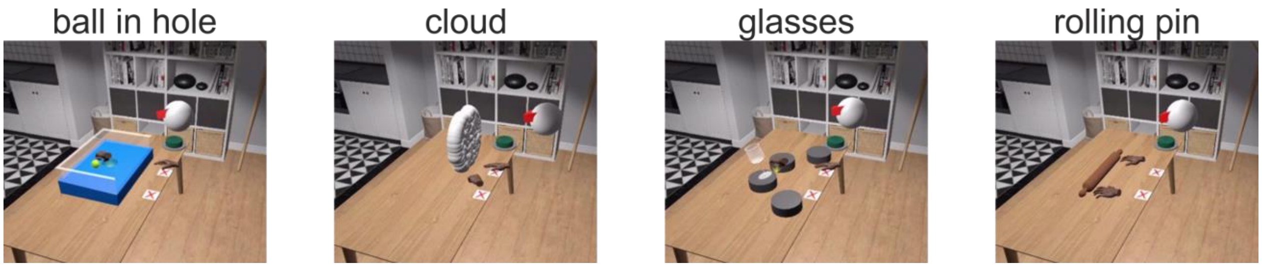

Figure S2 – The four tasks presently implemented in our VR system – *Ball in hole*: For this task, a box-like support with a pocket at its center is placed on the virtual table. At the beginning of each trial a tennis ball is placed on this support either to the right or left of the patients and they have to gently push the ball into the hole with their corresponding hand. *Cloud*: At the beginning of trial a cloud of small bubbles, which pop upon touching, appears. The cloud is placed either to the right or to the left of the patients and they have to pop all of the bubbles with the corresponding hand. *Glasses*: The task starts with four pedestals presented on the table. The pedestals are distributed along a circle centered on the patient's body at equal angular distances. A glass then appears on one randomly selected pedestal and the patients have to push it. *Rolling Pin*: In this task, the patients have to use both hands to push for a pre-defined distance a rolling pin on the table.

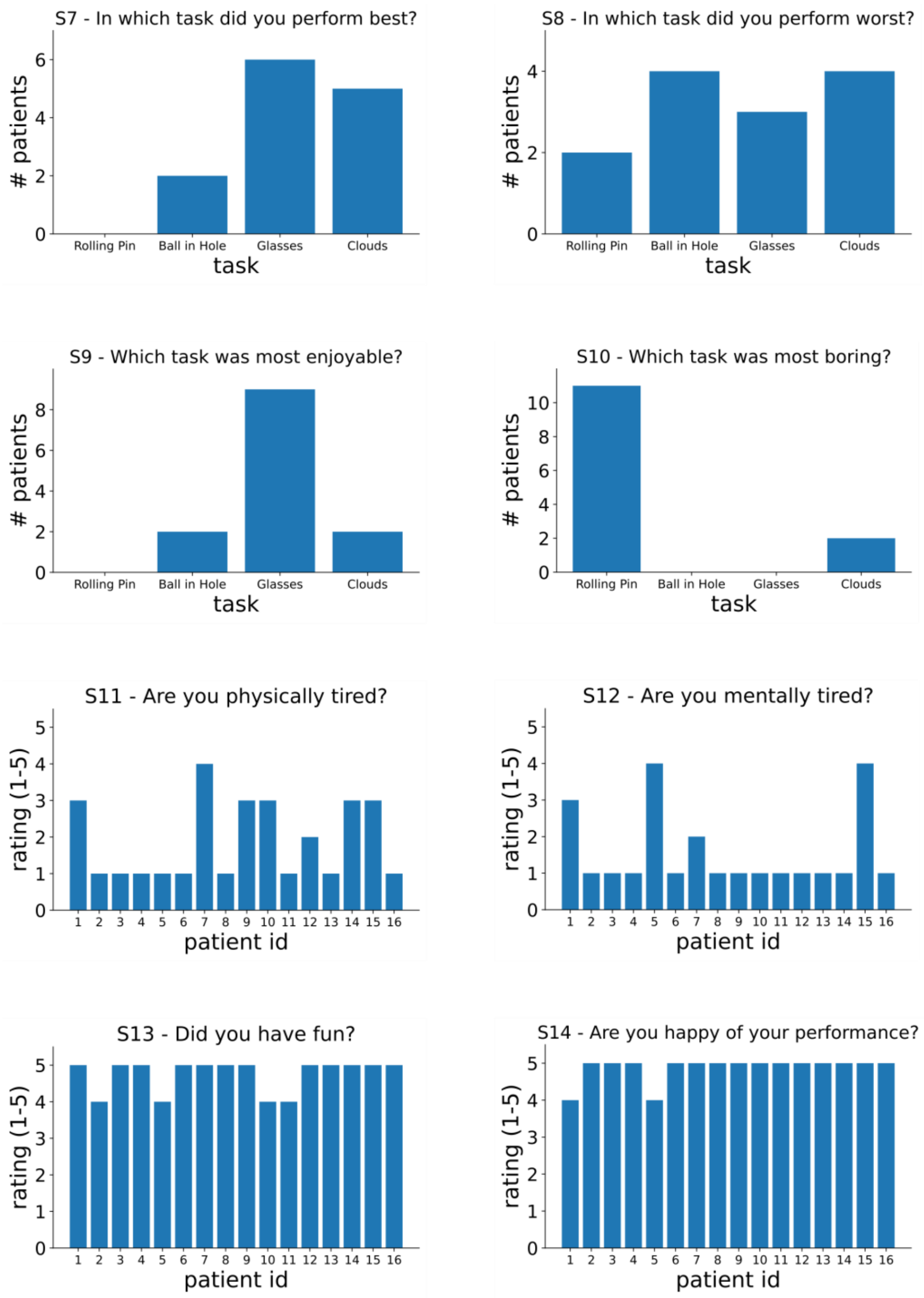

Figure S3 – Patients' responses to questions in the satisfaction questionnaire.

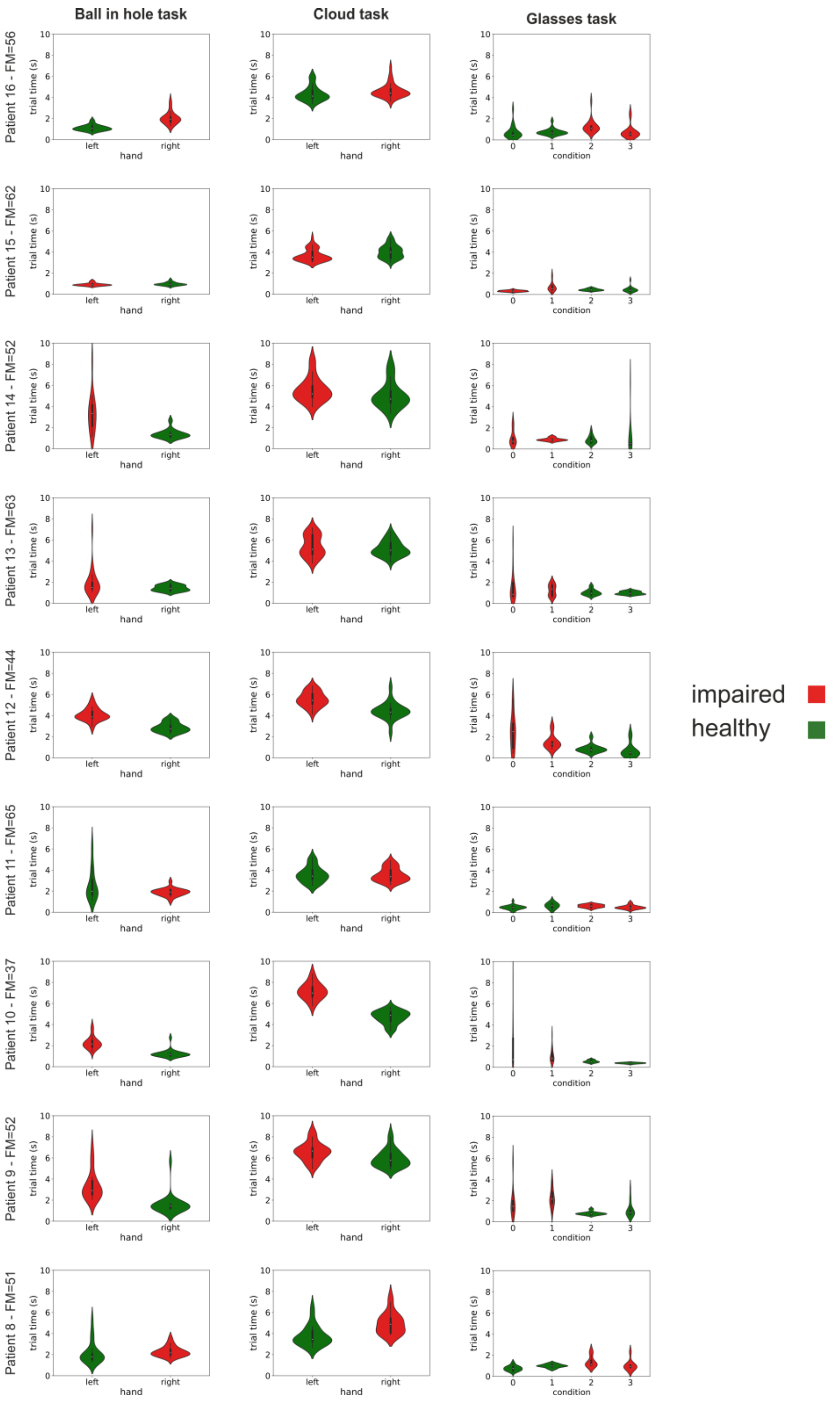

Figure S4 - Distribution of completion times for three tasks and all patients for which completion times were recorded (9 out of 16 patients).

References

Gonzalez-Franco, M., & Peck, T. C. (2018). Avatar embodiment. Towards a standardized questionnaire. *Frontiers in Robotics and AI*, *5*(JUN), 1–9. https://doi.org/10.3389/frobt.2018.00074
